# Efficient Anti-Fog and Anti-Reflection Functions of the Bio-Inspired, Hierarchically-Architectured Surfaces of Multiscale Columnar Structures

**DOI:** 10.3390/nano13091570

**Published:** 2023-05-07

**Authors:** Weixuan Li, You Chen, Zhibin Jiao

**Affiliations:** 1 College of Biological and Agricultural Engineering, Jilin University, Changchun 130022, China; 2 School of Mechanical Engineering, Shenyang University of Technology, Shenyang 110870, China

**Keywords:** bio-inspired hierarchically-architectured surfaces, multiscale columnar structures, anti-fog, anti-reflection, synergistic mechanism of anti-fogging and anti-reflection

## Abstract

Today, in the fields of optical precision instruments, medical devices, and automotive engineering, the demand for anti-reflection and anti-fog surfaces is growing rapidly. However, the anti-fog function often compromises the efficiency of the anti-reflection function. Therefore, optical precision instruments are always restricted by the inability to combine high anti-reflection efficiency and excellent anti-fog performance into one material. In addition, the synergistic mechanism of harmonizing anti-fogging and anti-reflection is currently unclear, which has a negative impact on the development and optimization of multifunctional surfaces. Herein, bio-inspired anti-fogging and anti-reflection surfaces (BFRSs) possessing multiscale hierarchical columnar structures (MHCS) were obtained using a brief and effective preparation technique, combining the biotemplating method and sol-gel method. Specifically, condensed fog droplets distributed on the BFRS can be absolutely removed within 6 s. In addition, the BFRSs endow the glass substrate with a relatively higher reflectance (17%) than flat glass surfaces (41%). Furthermore, we demonstrated the synergistic mechanism of the anti-fogging and anti-reflection functions of BFRSs. On the one hand, the high transparency benefits from the multiple refraction and scattering of light in the MHCS array. On the other hand, the excellent anti-fogging performance is attributed to the imbalance of the capillary force of the MHCS acting on the liquid film. The explanation for these two mechanisms provides more possibilities for the subsequent preparation of multifunctional surfaces. At the same time, the bionic research concept provides new solutions for the researcher to conquer the combination of high transmission and anti-fog properties for precision optical surfaces.

## 1. Introduction

The fogging of transparent surfaces has a serious impact on industrial production and our daily life, affecting surfaces such as automobile windshields, medical goggles, and near-sighted glasses, and especially precision optical surfaces [1,2,3]. Therefore, it is very urgent to develop transparent substrate surfaces with excellent anti-fogging functions. With the recent intensive research on the anti-fogging function of glass, various techniques and methods have been developed to design and fabricate various anti-fogging surfaces, such as the sol-gel method [4,5,6], spraying [7,8,9], chemical etching [10,11,12,13], plasma etching [14,15,16,17], and spin coating [18,19,20]. Although these surfaces have improved anti-fogging performance to some extent, there are still some problems that have not been solved, such as poor transmittance, complicated preparation processes, and severe environmental pollution. These problems seriously limit the wide application and practical popularity of anti-fogging surfaces.

Organisms in nature have evolved structures and functions that are perfectly adapted to their environment after brutal natural selection [21,22,23,24,25]. This provides a possible solution to the current anti-fog dilemma. Therefore, in recent years, researchers have conducted profound studies on typical organisms in nature and have been inspired and motivated by them to develop numerous super-wetting materials to achieve anti-fog functions [26,27,28,29], especially superhydrophilic materials [30,31]. Researchers have made significant progress in anti-fog performance, yet still have not solved the problem of low transmittance. In addition, the current bio-inspired superhydrophilic anti-fog materials are limited by their unclear anti-fog mechanism and unclear biomimetic mapping relationships [32,33].

It is worth noting that the butterfly *Trogonoptera brookiana* lives in the tropical rainforest with very high humidity in the air. The fog droplets condensed from the damp air are attracted to the wings of the butterfly, yet the butterfly wings are not affected by the fog droplets. In addition, butterfly wings must have low reflectivity in order to ensure the butterfly’s survival. Butterflies are poikilotherm, and therefore the heat of the sun is crucial to their survival. Therefore, butterfly wings possess polar reflectivity to store as much sunlight as possible. Consequently, butterfly wings give an inspired and promising approach to synergetic surfaces with anti-reflection and anti-fog functions. Niu et al. proposed a replicated butterfly wing structure to achieve fog resistance, but their fog resistance strategy has certain limitations and can only be achieved by diffusion through the anisotropic butterfly vein structure at both ends [34,35].

In this work, the anti-fog properties of the butterfly *Trogonoptera brookiana* wings were systematically investigated. Inspired by the special micro-nano structures of its wings, a bio-inspired surface with an anti-fogging function was successfully prepared on the surface of the glass substrate, using the biological template method and the butterfly wings. The surface consists of a single layer of silica, and the morphology and element distribution of the bionic surface was experimentally demonstrated. In addition, the super-wetting property of the bionic surface was confirmed by the characterization of the water contact angle test. More importantly, by conducting force analysis of water mist on the sample surface, the mechanism by which the water film was torn from the sample surface, and was destroyed and quickly removed from the surface, was obtained. Therefore, the bionic surface shows great potential value in various applications under practical conditions, such as medical endoscopes, medical protective glasses, optical precision instruments, etc. More importantly, the investigations in this work offer a promising way to usefully design and fabricate quasi-textured surfaces with multiscale hierarchical structures that possess high-performance physicochemical properties.

## 2. Materials and Methods

### 2.1. Materials

The butterfly *Trogonoptera brookiana* was selected as a bionic prototype and was obtained commercially from Dieyu Corporation (Shanghai, China). Before the experiments, the butterfly wings were shaken in ether and ethanol for 5 min and 7 min in sequence to remove the proteins and dust. Then, specimens were washed with deionized water and dried at room temperature for 1.5 h. Hydrochloric acid was provided by Sinopharm Chemical Reagent Co., Ltd., Beijing, China, and was diluted to 0.1M before use. Tetraethoxysilane (TEOS) was purchased from Aladdin Reagent Co., Ltd., Shanghai, China. In addition, diethyl ether, absolute ethyl alcohol, perchloric acid, and concentrated nitric acid were all purchased from Aladdin Reagent Co., Ltd., Shanghai, China. All chemicals were used as received without further purification.

### 2.2. Morphological Characterization, Chemical Composition Analysis, and Performance Testing

The surface morphology of butterfly wings and biomimetic samples were observed using a digital camera (EOS7D, CANON, Tokyo, Japan), a digital microscope (VHX-6000, KEYENCE, Tokyo, Japan), and a scanning electron microscope (SEM, JEOL Ltd., Tokyo, Japan). In addition, the chemical composition of butterfly wing scales and biomimetic samples were tested using EDS (equipped on SEM, JEOL Ltd., Tokyo, Japan), XRD (Bruker-AXS, Billerica, America. X-ray diffractometer system, recorded in the lower angle), and FTIR (FT/IR-6000, JASCO, Tokyo, Japan). The contact angles of butterfly wings, biomimetic samples, and ordinary glass were measured using a contact angle measuring instrument (JC2000A, Biolin Scientific, Espoo, Finland). The reflectivity of the butterfly wings, biomimetic samples, and ordinary glass were tested using a spectrometer (USB 4000, Ocean Optics, Dunedin, FL, USA), which was carefully calibrated using STD-WS. Fog droplets were generated at a speed of 30 cm·s^−1^ using an ultrasonic humidifier (YC-X100E, YADU, Beijing, China).

### 2.3. Preparation of Multiscale Hierarchical Honeycomb Structures (MHCS)

The preparation process of MHCS was via a simple inverted mold method. Initially, the clean butterfly wing was cut into small pieces measuring 3 cm in length and 2 cm in width using a scalpel. Then, the butterfly wing was sandwiched between two sheets of glass slides, which were rinsed with anhydrous ethanol and the ends of the glass were fixed with clips with moderate gripping force. In this experiment, the proper clamping force can make the precursor solution enter the interspace between the slides and come into contact with the butterfly’s microscopic surface texture. The position between the glasses was fixed to prevent bionic reverse structure distortion. Next, ethyl orthosilicate (TEOS) was mixed with 0.1 M hydrochloric acid solution at a volume ratio of 3:1 and stirred with a magnetic agitator until the solution changed from cloudy to clear, to obtain the precursor solution. The 5 μL precursor solution was injected into the glass and diffused through the whole wings via capillary force, to fill the honeycomb structure. Then, the above components were placed in an air-dry oven at 125 °C for 30 min. The high temperature accelerated the gel process of the precursor solution. In addition, 100 mL of the perchloric acid and 100 mL of concentrated nitric acid were added to the breaker. The solution was injected into the petri dish, which was placed in an air-dry oven with a 130 °C temperature and a 30-min heating time to dissolve the chitin of the butterfly wing surface with the strongly acidic and oxidizing solution. The entire assembly was cleaned in deionized water by ultrasonic oscillation for 5 min to remove any residue. Finally, the anti-structure was obtained after drying at room temperature.

## 3. Results

### 3.1. Biological Templates and General Characteristics of Butterfly Wing Scales

The butterfly is one of the most popular bionic prototypes today, due to its many excellent characteristics. In this work, the wings of the butterfly *Trogonoptera brookiana* was used as a biological model. This species inhabits the tropical rainforest at elevations between 500–1500 m above sea level. The butterfly has evolved anti-fogging structures on wings for its typical foggy and humid habitat. The characteristics of ectotherm require butterfly wings to reduce reflectivity to absorb as much energy from the sun as possible. This provides inspiration for the design of anti-fog and anti-reflective surfaces.

**Figure 1:**
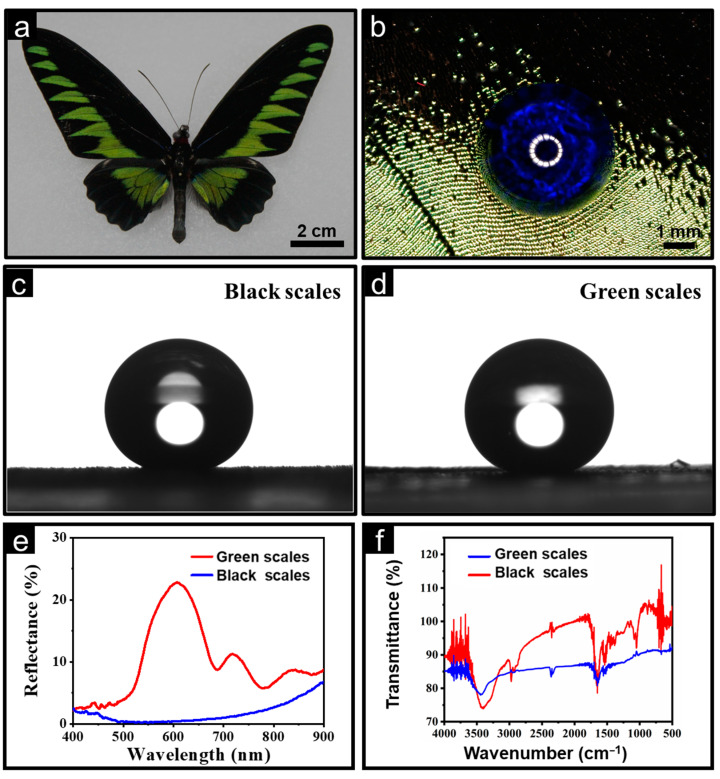
Characteristics of butterfly wings. (**a**) Overall view of the butterfly. (**b**) Water contact angles of the butterfly wing. (**c**) Water contact angles of black scales. (**d**) Water contact angles of green scales. (**e**) The reflectance of the butterfly wings. (**f**) FTIR spectra analysis of butterfly wings.

The digital photo of the original butterfly *Trogonoptera brookiana* is shown in Figure 1a. The wingspan of the butterfly was approximately 14 cm and the forewing was draped with neatly arranged long, smooth black strips, which looked as beautiful as black velvet. Afterwards, the water-repellent and anti-fogging capability of the butterfly wings were evaluated by examining the wattability of droplets with the help of the camera and contact angle (CA) measuring instrument. The water droplets keep the morphology of the sphere, as shown in Figure 1b, and the water CAs of the black and green regions were 152.7° and 153.4°, respectively (Figure 1c,d). This indicates that the butterfly wings preventing water infiltration to their surface is responsible for the water-repellent and anti-fogging capability. Reflectance spectroscopy, ranging from 400 nm to 900 nm, was performed on the butterfly wings, as shown in Figure 1e. The butterfly wings show a strong absorption peak near 595 nm, as seen by the reflection spectrum of the green region of the butterfly wings. This is consistent with the color expression of the butterfly wings themselves. In addition, the comparison shows that the black area of the butterfly wing has a lower reflectivity than the green area, and thus the black part was selected for subsequent testing, given that it has a reflectivity of less than 7%. The black regions of the butterfly were chosen as the experimental regions to be studied carefully. Furthermore, the Fourier transform infrared spectrum (FTIR) was employed to determine the chemical composition of the butterfly wings, and the test results are shown in Figure 1f. The absorption peak of about 3430 and 3270 cm^−1^ corresponds to the tensile vibration of the hydroxyl and N-H bonds. The absorption peaks near 2980, 2920, and 2880 cm^−1^ are formed by the tensile vibration of the hydrocarbon bond. The absorption vibration peak of carbon dioxide appears at 682 and 2360 cm^−1^. The 1660, 1552, and 1320 cm^−1^ peaks located near the fingerprint region are the absorption peaks of amide I, amide II, and amide III, respectively. The weak peak around 885 cm^−1^ was assigned to the stretching vibration of the hexatomic ring. Amide groups and six-atom rings together form the skeleton component of the butterfly wing, chitin.

In conclusion, based on the above analysis, it can be concluded that the scale is mainly composed of chitin and organic components. The water-insoluble characteristics of chitin and organic components, coupled with the micro/nano rough structure of the butterfly wing surface, make the butterfly wing exhibit excellent superhydrophobic performance, anti-fog, and anti-reflection properties.

### 3.2. Microscopic Morphology Observations and Chemical Composition Analysis of Butterfly Wing Scales

**Figure 2:**
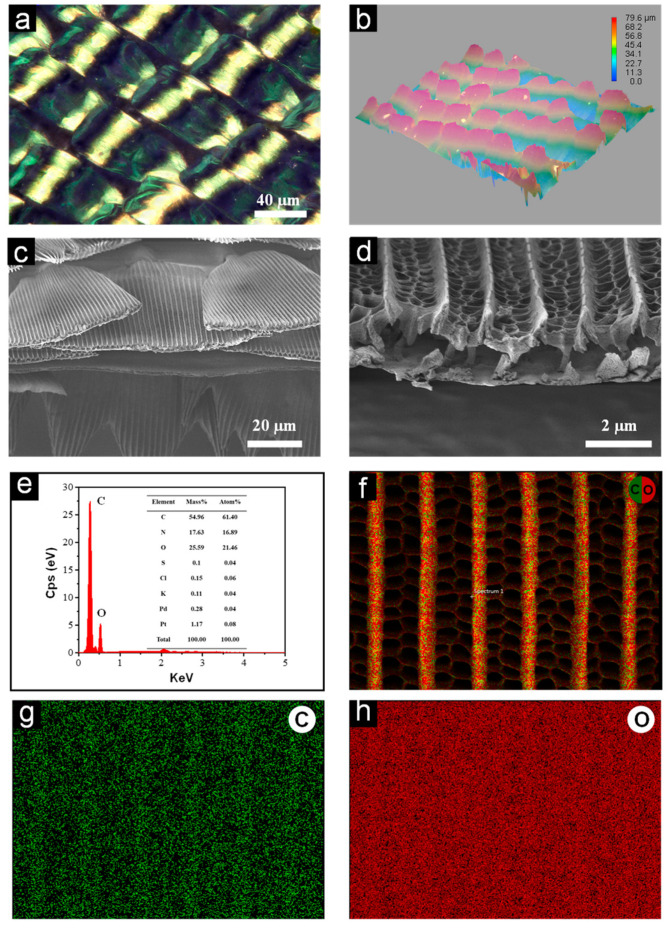
Composition and structure analyses of butterfly wings. (**a**) Stereoscopic microscopy image of the butterfly wings. (**b**) The roughness of the butterfly wings. (**c**,**d**) SEM images of the wing scales. (**e**–**h**) EDS spectrum analysis.

With the aid of an optical microscope, we can clearly discover that the black region of a butterfly’s wing is actually composed of black scales. These scales overlap and interlace with each other, similar to shingle distribution. Specifically, a single scale measures around 50 μm in width and 150 μm in length. The roughness of the entire butterfly wing was observed using the ultra-depth, three-dimensional microscope, and the test results are shown in Figure 2b. It is evident from the image that the end of a single layer of scales overlaps the upper end of the lower layer of scales. The roughness of the entire wings was measured to be 79.6 μm. In order to better understand the laying rules of each layer of butterfly wing scales, the micro/nano structures and arrangement rules of the butterfly wing surface were observed in more depth using FESEM. As can be seen from Figure 2c, the root of the scale is embedded in the substrate and extends outward. Moreover, finer nanostructures such as ridges and quasi-honeycomb-like structures were observed as shown in Figure 2d. There were parallel fold stripes on both sides of the ridges. Interestingly, the periodic space between adjacent ridges was filled with a netlike reticulum composed of pores, showing a typical “honeycomb” shape. The height of the longitudinal ridges is approximately 1.9 μm. It is worth noting that all black scales have similar multiscale hierarchical structures, including ridges, reticulum, and stripes. Herein, we named these complex structures as multiscale hierarchical honeycomb structures (MHCS). Subsequently, an EDS analysis was performed on the butterfly wings, and the results are shown in Figure 2e–h. As could be seen from the elemental content, it is evident that the major elements are carbon (C), oxygen (O), and nitrogen (N). The atomic percentages of these three elements were 61.40%, 16.89%, and 21.46%, respectively.

### 3.3. The Preparation Process of the BFRSs

**Figure 3:**
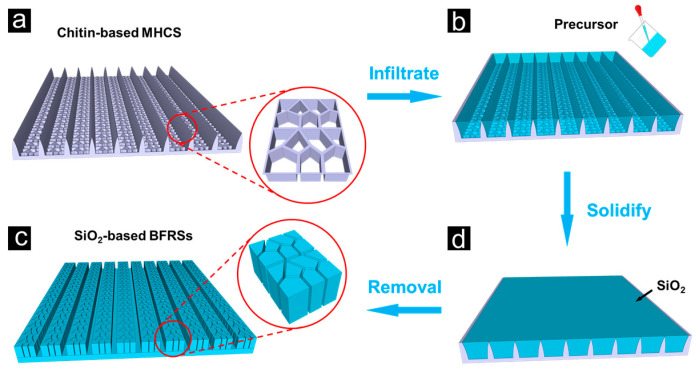
The fabrication process of the MHCS-based BFRSs. (**a**) the butterfly wings were washed with ether, ethanol, and deionized water to eliminate any surface grease from the butterfly wings, and then the clean part of the butterfly wings was sandwiched between two slides with the other part exposed to the air; (**b**) 10 μm of the precursor solution was put onto the exposed surface of the butterfly wings using a pipette gun; (**c**) the whole sample was moved to the oven and heated at 125 °C for 50 min; (**d**) after the sample was cured, it was again placed in the oven at 250 °C for 120 min to remove the original stencil from the surface at a high temperature.

Figure 3 displays the overall preparation process using a 3D model. The samples were then sonicated and shaken for 10 min to remove the residues from the surface. The BFRSs were obtained after being dried at room temperature.

### 3.4. The Preparation Process of the BFRSs

**Figure 4:**
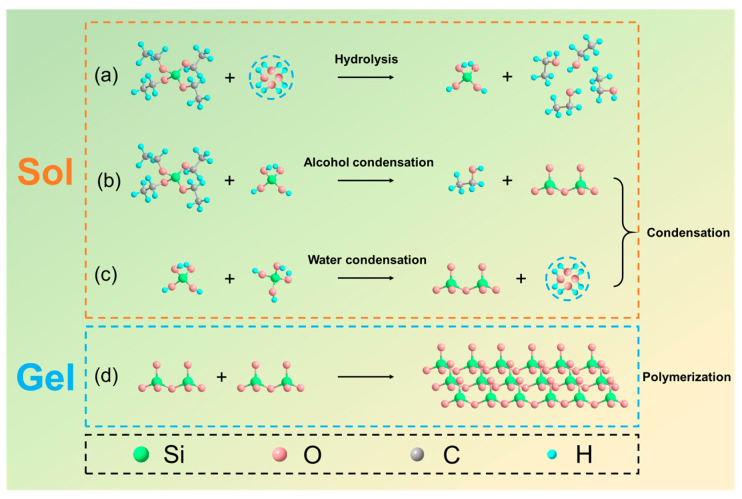
Reaction mechanism of the BFRSs. (**a**–**c**) Hydrolysis and condensation reaction of TEOS. (**d**) Polymerization reaction of SiO_2_.

The corresponding internal chemical synthesis mechanism was described at the functional group level in Figure 4 [34,36]. It is evident from Figure 4a–d that the 3D SiO_2_ networks making up the biomimetic MHCS evolve sequentially as the product of successive hydrolysis and condensation reactions (and the reverse reactions, esterification, and alcoholic or hydrolytic depolymerization). The hydrolysis reaction and condensation reaction constitute the primary processes of the overall reaction. The precursor solution filled the gap between the butterfly wing and the glass sheet and was cured by heat treatment. After the original biotemplate material was removed through high-temperature ablation, MHCS was obtained, which further forms the expected BFRS.

### 3.5. Microscopic Morphology Observations and Chemical Composition Analysis of the BFRSs

**Figure 5:**
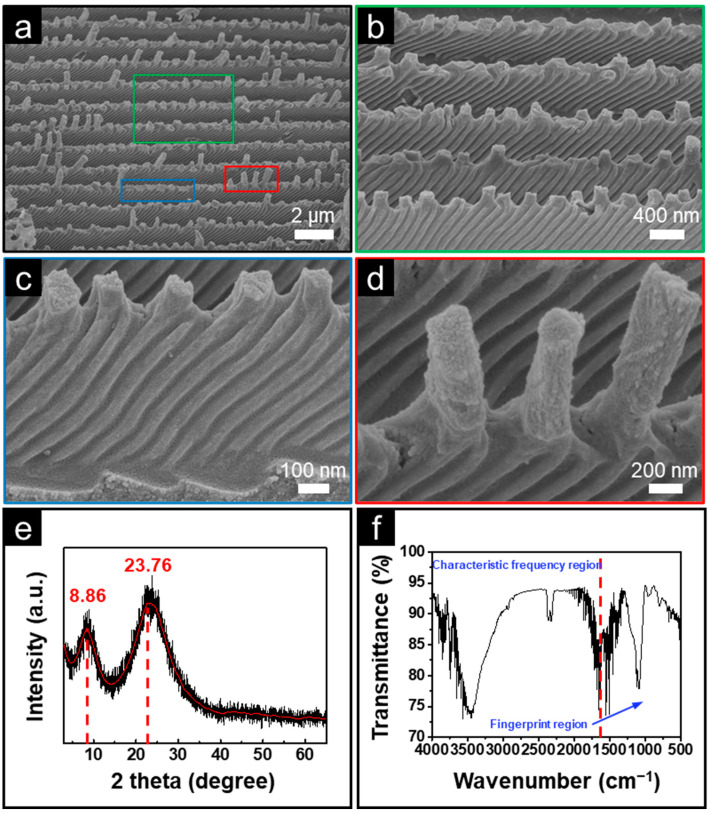
Characteristic analysis of BFRSs. (**a**–**d**) Microscopic morphology of the BFRSs. (**e**) The XRD spectra of BSM. (**f**) The FTIR spectra analysis of BFRSs.

The micro/nano structures of BFRSs were observed using FESEM, and the observation results are shown in Figure 5a. Figure 5a shows the structural details at a single scale. It can be seen that the BFRSs consist of many ridges with a monopolistic structure and many grooves at the base of these ridges, which provide natural conditions for the BFRSs to be anti-reflective. Further magnification shows that there are many columnar structures at the top of the spine vein structure and the height of the columnar structures is not uniform. Figure 5c,d show that the grooves are about 15 nm wide and do not intersect with each other. The top of the groove is connected to the column structure. This channel facilitates water flow evacuation after the tearing of the water film, resulting in the anti-fog function. By observing the BFRSs, we can see that the biological structures are better replicated. Predictably, the good light-trapping and housing structures of the butterfly wings themselves are also successfully replicated. Figure 5e shows the XRD spectrum of BFRSs. It can be seen that a characteristic peak of silicon dioxide appears at 23.76, indicating that the reaction was successfully carried out and silicon dioxide was formed. As shown in Figure 5f, the absorption peak near 1100 cm^−1^ is the anti-symmetric stretching vibration peak of Si-O-Si. The peak near 800 cm^−1^ is the symmetric stretching vibration peak of the silicon–oxygen bond. The broad peak near 3450 cm^−1^ is the anti-symmetric stretching vibration peak of the hydroxyl group in structural water. Furthermore, the peak near 1650 cm^−1^ is the bending vibration peak of H-O-H in water, and the peak near 955 cm^−1^ is the absorption peak of the bending vibration of Si-OH. This indicates that silicon dioxide was successfully generated in the reaction and confirms the chemical composition of BFRSs.

### 3.6. Anti-Reflection and Anti-Fog Capability of BFRSs

**Figure 6:**
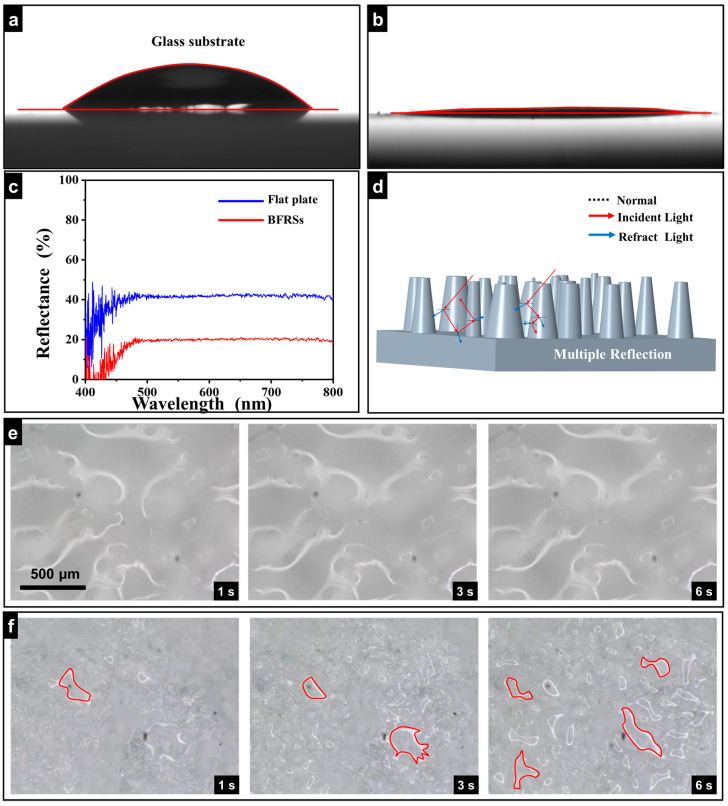
Anti-reflection and anti-fog test. (**a**) The water contact angle of the glass substrate. (**b**) The water contact angle of the BFRSs. (**c**) The reflectance analysis of the flat plate and BFRSs. (**d**) Anti-reaction mechanism analysis of the BFRSs. (**e**) Anti-fog test of the flat plate. (**f**) Anti-fog test of the BFRSs.

In the process of characterizing the fog-collecting performance of the bionic structure, it was found that the bionic structure increases the transmittance of the glass sheet. In addition, the presence of the bionic structure also results in the glass surface being superhydrophilic. In order to further characterize the optical properties of the biomimetic column structure, the reflectance of both biomimetic structure replicas and smooth slides was measured using a fiber optic spectrometer. As shown in Figure 6c, the maximal reflectance values of glass are about 41% in the range of 400 nm and 800 nm. However, the maximum reflectivity of the BFRSs is about 17%, which accounts for 2/5 of the glass transmittance. To explain this phenomenon, we drew a simplified model of the bionic columnar structure array to explain its multiple anti-reflection mechanisms in Figure 6d. When the incident light propagates through the air to the surface of the silica columnar structure, scattering occurs at the interface between the air and the solid, which includes both reflection and refraction. It can be found, from the optical path diagram drawn in the figure, that light has been reflected and refracted many times in the columnar array. Multiple reflections increase the distance that light travels in the columnar array structure, thus increasing the probability that light will collide with the columnar structure, and multiple refractions also enhance the absorption of light energy within the columnar structure. Both of these methods enhance the efficient absorption of solar energy by the columnar array structure, with only a relatively small amount reflected back into the air. In addition, the nanoscale convex hull distributed on the side of the columnar structure enhances the light scattering of the bionic anti-structure replica, further reducing the light reflection. The superhydrophilic property of BFRSs is the core attribute of their superhydrophilic fog-collecting behavior. The superhydrophilic property endows the BFRSs with the strong ability to capture water molecules. To characterize the superhydrophilic properties of replicas, a contact angle meter was used to evaluate the wetting behavior of water droplets on the surface. As the control group, the intrinsic contact angle was measured on the untreated slide surface. As shown in Figure 6a, water droplets arched on the untreated slide, and the contact angle was 48.4°. The characteristic contact angle of water droplets on the anti-structure BFRSs’ surface was approximately 6° (Figure 6b), which proves their superhydrophilic property. Figure 6e,f show the fog collection behavior of the BFRSs, with the whole surface being almost transparent 1.0 s after the end of the spray, indicating that the surface is completely permeable.

For comparison, within 1.0 s after the end of the spray, several ellipsoidal droplets condensed on the surface of the untreated slide. These droplets were evenly distributed on the surface. As the spraying process continued, these droplets gradually grew and diffused. Due to the slide surface not being superhydrophilic, the surface tension of water droplets and the cohesion of internal water molecules made it difficult for water droplets to connect and form a liquid film. As a result, the fog collection efficiency on the slide surface was greatly reduced, and this effect did not change significantly as time progressed. At 6 s after the end of the spray, the liquid film on the BFRSs was torn and flowed away quickly. This proves that the BFRSs have good fog collection ability.

### 3.7. Autonomous Fog-Collecting Mechanism of BFRSs

**Figure 7:**
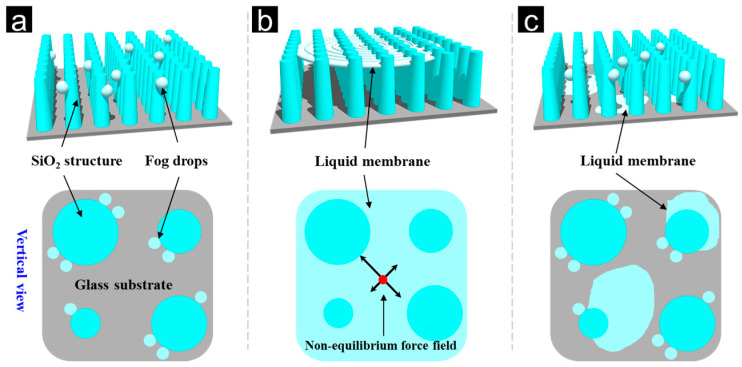
Anti-fog mechanism analysis of BFRSs. (**a**) Fog capture stage. (**b**) Water film tearing stage. (**c**) Fog removal stage.

Figure 7 illustrates the autonomous fog-collecting mechanism of BFRSs based on the morphological observation results of FESEM, which was used to construct a 3D visualization model of the bionic cylindrical array structure according to the characteristic parameters of the columnar array structure. On the basis of this model, mechanical analysis and surface energy theory were applied to the dynamic process of autonomous fog collection. According to the results of transmittance spectrum measurement and high-speed frequency observation, the active fog-collecting behavior of BFRSs is divided into two stages. The first stage is fog capture, during which water molecules are adsorbed by bionic column array and gradually form a water film on the top. When the water film is formed, it can absorb water molecules in the air more quickly and expand to the whole bionic surface. The second stage is the water film tearing stage. The bionic cylindrical array attached to the water film is not uniform, and therefore the force of the cylindrical structure received by the water film in each area is different. These uneven forces eventually tear the liquid film, which becomes droplets and flows away from below. The existence of these two stages ensures the anti-fogging efficiency of the BFRSs.

## 4. Conclusions

In summary, BFRSs with MHCS were prepared through a brief and effective technique combing the biotemplating method and sol-gel method. Specifically, the MHCS was inspired by the feature architectures of butterfly wings. The morphology and elemental composition of the BFRSs were analyzed using FESEM and EDS, and the results showed that the BFRSs perfectly inherited the structural features of the butterfly wings, and Si and O were uniformly distributed on the surface of the BFRSs. Subsequently, the composition of BFRSs was further verified using XRD and FTIR. Finally, the BFRSs were tested for their anti-reflection performance and anti-fog performance. Remarkably, condensed fog droplets distributed on the BFRS can be absolutely removed within 6 s. In addition, the BFRS endows the glass substrate with a relatively higher reflectance (17%) than flat glass surfaces (41%). Most importantly, the anti-fogging and anti-reflection mechanism of the bionic surface is described in detail. The proposed design strategy can be easily and stably implemented in a wide range of practical applications, in mirror anti-fog, and urban light pollution.

## Data Availability

Data will be made available on request.
